# Prostaglandin‐cytokine crosstalk in chronic inflammation

**DOI:** 10.1111/bph.14530

**Published:** 2018-12-18

**Authors:** Chengcan Yao, Shuh Narumiya

**Affiliations:** ^1^ Centre for Inflammation Research, Queen's Medical Research Institute The University of Edinburgh Edinburgh UK; ^2^ Alliance Laboratory for Advanced Medical Research and Department of Drug Discovery Medicine, Medical Innovation Center Kyoto University Graduate School of Medicine Kyoto Japan

## Abstract

Chronic inflammation underlies various debilitating disorders including autoimmune, neurodegenerative, vascular and metabolic diseases as well as cancer, where aberrant activation of the innate and acquired immune systems is frequently seen. Since non‐steroidal anti‐inflammatory drugs exert their effects by inhibiting COX and suppressing PG biosynthesis, PGs have been traditionally thought to function mostly as mediators of acute inflammation. However, an inducible COX isoform, COX‐2, is often highly expressed in tissues of the chronic disorders, suggesting an as yet unidentified role of PGs in chronic inflammation. Recent studies have shown that in addition to their short‐lived actions in acute inflammation, PGs crosstalk with cytokines and amplify the cytokine actions on various types of inflammatory cells and drive pathogenic conversion of these cells by critically regulating their gene expression. One mode of such PG‐mediated amplification is to induce the expression of relevant cytokine receptors, which is typically observed in Th1 cell differentiation and Th17 cell expansion, events leading to chronic immune inflammation. Another mode of amplification is cooperation of PGs with cytokines at the transcription level. Typically, PGs and cytokines synergistically activate NF‐κB to induce the expression of inflammation‐related genes, one being COX‐2 itself, which makes PG‐mediated positive feedback loops. This signalling consequently enhances the expression of various NF‐κB‐induced genes including chemokines to macrophages and neutrophils, which enables sustained infiltration of these cells and further amplifies chronic inflammation. In addition, PGs are also involved in tissue remodelling such as fibrosis and angiogenesis. In this article, we review these findings and discuss their relevance to human diseases.

**Abbreviations**
AD: atopic dermatitis
APC: antigen‐presenting cell
AS: ankylosing spondylitis
CBP: CREB binding protein
CD: Crohn's disease
CREB: cAMP response element binding protein
CRTC2: CREB regulated transcription co‐activator 2
DAMP: damage‐associated molecular pattern
DC: dendritic cells
EAE: experimental autoimmune encephalomyelitis
FLS: fibroblast‐like synoviocyte
GC: germinal centre
GWAS: genome‐wide association study
IA: intracranial aneurysm
IBD: inflammatory bowel disease
ILC: innate lymphoid cell
ILC1: type 1 ILC
ILC2: type 2 ILC
ILC3: type 3 ILC
IP: PGI receptor
KO: knockout
mPGES1: microsomal PGE synthase‐1
MS: multiple sclerosis
NSAID: non‐steroidal anti‐inflammatory drug
OVA: ovalbumin
PAMP: pathogen‐associated molecular pattern
RA: rheumatoid arthritis
TARC: thymus and activation‐regulated chemokine
TCR: T‐cell receptor
Th cell: helper T‐cell
Th1 cell: type 1 Th cell
Th17 cell: type 17 Th cell
Th2 cell: type 2 Th cell
TLR: toll‐like receptor
Treg: regulatory T cell

## Introduction

Upon invasion of foreign pathogens or tissue damage, the innate immune system is immediately activated in response to molecules bearing pathogen‐associated molecular patterns (PAMPs) and damage‐associated molecular patterns (DAMPs), recruits granulocytes to the injured tissue to clear pathogens, produces inflammatory mediators, including pro‐inflammatory cytokines such as TNF‐α, IL‐1β and IL‐6 and lipid mediators such as PGs and leukotrienes (LTs), and evokes an acute inflammatory process (hours to days) to clear the pathogens and damaged tissues. Acute inflammation is resolved and the tissue is repaired when PAMPs, DAMPs, pathogens and damaged tissues are cleared, granulocyte recruitment ceases with a down‐regulation and scavenging of chemokines, and recruited granulocytes are subsequently cleared by efferocytosis. However, inflammation often becomes chronic (weeks to months to years), and this underlies various chronic disorders such as autoimmune, neurodegenerative, vascular and metabolic diseases and cancer. Recent studies in various experimental systems have begun to unravel the possible mechanisms through which inflammation is sustained and becomes chronic. They include the generation of positive feedback mechanisms that self‐amplify inflammatory responses and the suppression of negative feedback mechanisms that prevent resolution, which leads to the recruitment, activation, phenotypic transformation and synergistic interaction of various types of cells and sustains pro‐inflammatory cytokine signalling at inflammatory sites.

**Figure 1:**
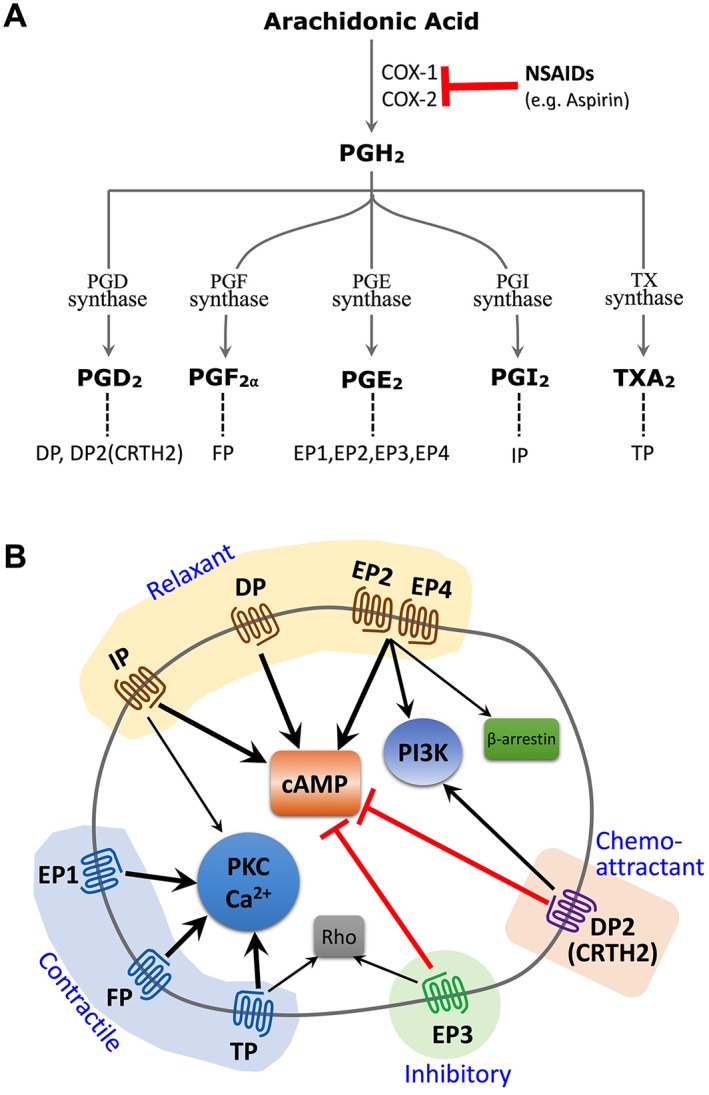
PG biosynthesis, receptors and signalling pathways. (A) Arachidonic acid is metabolized by COX, either COX‐1 or COX‐2, to PGH_2_, which is then converted to each PG, PGD_2_, PGE_2_, PGF_2⍺_, PGI_2_ or TXA_2_, by respective synthases. NSAIDs inhibit COXs and suppresses PG biosynthesis. Each PG acts on its cognate receptor to exert its actions. PGD_2_ acts on PGD receptors, DP_1_ and DP_2_ (formerly designated as CRTH2, chemoattractant receptor‐homologous molecule expressed on Th2 cells); PGE_2_ acts on four subtypes of PGE receptors, EP_1_, EP_2_, EP_3_ and EP_4_; PGF_2α_ acts on PGF receptor, FP; PGI_2_ acts on PGI receptor, IP; TXA_2_ acts on TXA receptor, TP. (B) All of the nine PG receptors are GPCRs and exert their actions by modulating second messengers and intracellular signal transduction. Dependent on their downstream signalling pathways and structural similarity, they are classified into three groups. The relaxant group consists of DP_1_, EP_2_, EP_4_ and IP that are mainly coupled to an elevation of intracellular cAMP levels and activate the cAMP‐PKA‐CREB pathway. The contractile group consists of EP_1_, FP and TP that are coupled to Ca^2+^ signalling and PKC activation. Both EP_3_ and DP_2_ receptors are coupled to a reduction in the levels of intracellular cAMP and belong to the inhibitory PG receptor group. However, while EP_3_ receptors with other PG receptors constitute the family of PG receptors, DP_2_ belongs to the chemo‐attractant GPCR family. In addition to these main downstream signalling pathways, each PG receptor also activates other signal transduction pathways. For example, EP_2_ and EP_4_ receptors activate the PI3K pathway and form a complex with β‐arrestin to transactivate the EGF receptor; TP and EP_3_ receptors activate the small G‐protein Rho; IP activates the PKC and Ca^2+^ signalling; and DP_2_ receptors activate the PI3K pathway. Furthermore, EP_3_ receptor has various alternatively spliced variants in its carboxyl terminus, which can couple to various signalling pathways other than the pathways mentioned (Sugimoto and Narumiya, 2007).

PGs including PGD_2_, PGE_2_, PGF_2α_, PGI_2_ and TXA_2_ are produced in most tissue and cells either constitutively by physiological stimuli or in response to noxious stimuli. In either case, C20‐unsaturated fatty acids such as arachidonic acid are released from phospholipids in the cell membrane and converted into PGH_2_ by cyclooxygenases (COXs including COX‐1 and COX‐2). PGH_2_ is then converted into each PG by respective PG synthases (Figure 1A). PGs exert their actions through a family of eight types and subtypes of GPCRs, PGD receptor (originally named DP and now called DP_1_), EP_1_, EP_2_, EP_3_ and EP_4_ subtypes of PGE receptor, PGF (FP) receptor, PGI (IP) receptor and TXA (TP) receptor and another PGD receptor in a different GPCR family, originally named chemoattractant receptor‐homologous molecule expressed on Th2 cells (CRTH2) and now called DP_2_ receptor. These PG receptors activate distinct downstream signalling pathways and thus have divergent, sometimes additive and other times opposing, functions in various physiological and pathological processes. For example, while EP_2_, EP_4_, DP_1_ and IP receptors activate cAMP signalling, EP_3_ and DP_2_ receptors inhibit cAMP signalling. EP_1_, FP and TP receptors mainly activate the PKC and Ca^2+^ pathways. TP and EP_3_ receptors also activate the small G‐protein Rho; EP_2_ and EP_4_ receptors can also activate PI3K and β‐arrestin pathways (Figure 1B). Aspirin‐like non‐steroidal anti‐inflammatory, anti‐pyretic and analgesic drugs (NSAIDs) exert their actions by targeting COX and inhibiting PG biosynthesis. PAMPs/DAMPs such as LPS and pro‐inflammatory cytokines such as IL‐1β and TNF‐α induce the expression of inducible isoforms of COX and PGE synthase, COX‐2 and microsomal PGE synthase 1 (mPGES1) respectively (Díaz‐Muñoz *et al*., 2010). Therefore, PGs have been traditionally thought of as inflammatory mediators linking innate immunity to acute inflammation. Indeed, studies using genetically modified mice deficient in each PG receptor identified types of PG and their receptors involved in each of the acute inflammatory responses (Narumiya and Furuyashiki, 2011). However, expression of COX‐2 and mPGES1 is not limited to the sites of acute inflammation but widely seen in tissues of chronic inflammation such as the joints of rheumatoid arthritis (RA) patients, the spinal cord of multiple sclerosis (MS) patients, the colon of inflammatory bowel disease (IBD) patients, the cerebral arterial wall of cerebral aneurysm patients and tumours and their micro‐environment of many cancers (Ricciotti and FitzGerald, 2011; Wang and DuBois, 2018). These findings suggest that in addition to their actions in acute inflammation, PGs also play important roles in many chronic inflammatory diseases. Recent studies using PG receptor knockout (KO) mice and PG receptor‐type selective agonists and antagonists in various animal models of chronic diseases have revealed that PGs intimately crosstalk with cytokines, drive pathogenic conversion, recruit and activate inflammatory cells and contribute to chronic inflammation through various mechanisms. Furthermore, genome‐wide association studies (GWASs) and gene signature analyses of disease tissues of patients strongly suggest the relevance of these experimental findings to chronic inflammation in humans. Here, we review these findings and discuss the therapeutic potential of PG‐related drugs in chronic human diseases.

## PG‐cytokine crosstalk in immune and allergic inflammation

**Figure 2:**
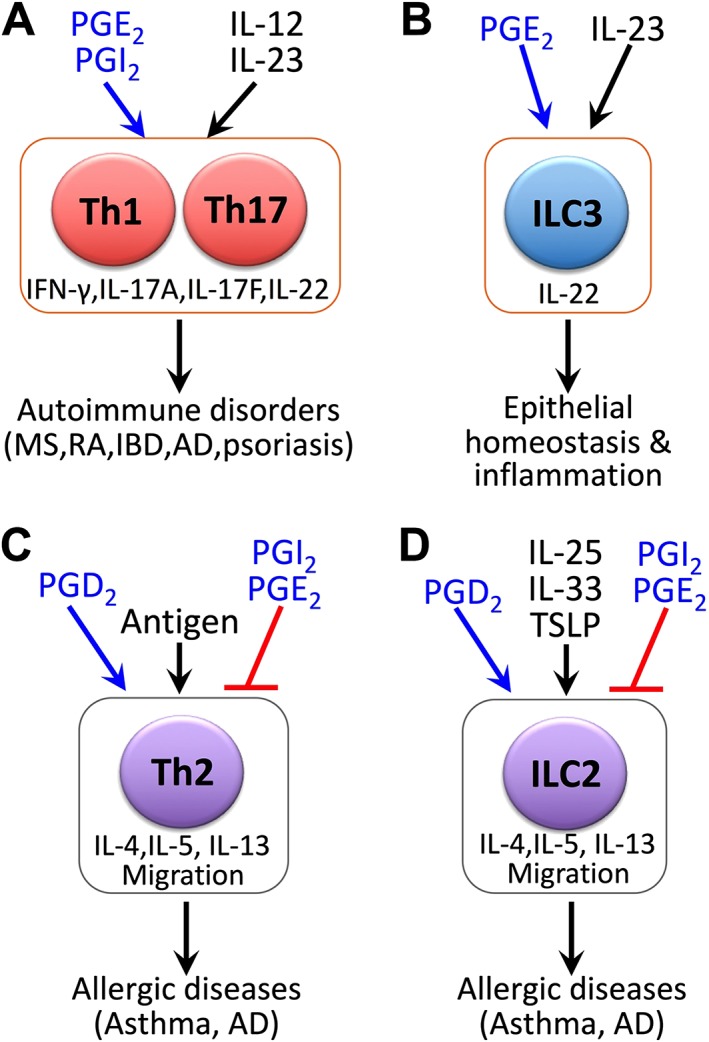
PG‐cytokine crosstalk in the regulation of lymphocyte‐mediated chronic inflammatory responses. (A) PGE_2_ and PGI_2_ crosstalk with IL‐12 and IL‐23 signalling pathways in Th1 and Th17/Th22 cells, respectively, and promote differentiation and expansion of these Th cell subsets both *in vitro* and *in vivo*, producing inflammatory cytokines such as IFN‐γ, IL‐17A, IL‐17F and IL‐22. These PG actions underlie Th1 and Th17 cell‐mediated autoimmune inflammation in mouse models of MS, RA, IBD, atopic dermatitis (AD) and psoriasis. (B) In response to IL‐23 stimulation, type 3 innate lymphoid cells (ILC3) produce the cytokine IL‐22, critical to epithelial homeostasis and modulation of inflammation. PGE_2_ has been shown to augment IL‐23‐induced IL‐22 production from ILC3s through the EP4‐cAMP‐PKA pathway, facilitating intestinal epithelial homeostasis and control of systemic inflammation. (C) PGE_2_ and PGI_2_ inhibit antigen‐dependent activation of Th2 cells and production of type 2 cytokines (e.g. IL‐4, IL‐5, IL‐13) and thus limit type 2 allergic inflammation. In contrast, PGD_2_ promotes allergic inflammation through DP_2_ receptor‐mediated Th2 cell migration. (D) Epithelial cytokines such as IL‐25, IL‐33 or thymic stromal lymphopoietin (TSLP) stimulate ILC2 to produce type 2 cytokines, developing type 2 allergic immune diseases such as asthma and AD. While PGD_2_ promotes type 2 allergic immune responses through driving ILC2 cell migration *via* DP_2_ receptors, PGI_2_ and PGE_2_ inhibit allergic immune responses through suppressing epithelial cytokine‐induced production of type 2 cytokines from ILC2 cells.

Acute inflammation often becomes chronic, when acquired immunity is raised against pathological antigens generated at inflammatory sites and causes immune inflammation. Antigens generated at inflammatory sites are taken up by antigen‐presenting cells (APCs), such as dendritic cells (DCs) and macrophages, which are activated upon this uptake, migrate to draining lymph nodes and present the antigens to T lymphocytes to trigger the adaptive immune system. Activated APCs also produce various cytokines, which prime antigen‐activated T‐cells to differentiate into specific subsets of helper T (Th) cells that produce the subset‐specific cytokines and trigger inflammation. The type 1 subset of Th cells (Th1) produce IFN‐γ, Th2 cells produce type 2 cytokines including IL‐4, IL‐5 and IL‐13 and Th17 cells produce IL‐17A, IL‐17F and IL‐22. The generation of Th1, Th2 and Th17 cells is primarily to expel invaded pathogens, but activation and differentiation of these T‐cells, if not properly controlled, can trigger immune diseases (Zhu *et al*., 2010). Th1 and Th17 cells play critical roles in the development of autoimmune inflammatory diseases such as MS, IBD, RA and psoriasis. Indeed, the accumulation of Th1 and Th17 cells and elevation of their signature cytokines have been found in the brain, synovial fluid, gut and skin of patients with MS, RA, Crohn's disease and psoriasis respectively (Zhu *et al*., 2010). Furthermore, administration of antibodies targeting the cytokines they produce has shown clinical efficacy in some of these diseases (Zhu *et al*., 2010). In contrast, Th2 cells are involved in allergic inflammation such as atopic dermatitis (AD) and asthma. As described below, a number of studies now suggest that PGs, particularly PGE_2_, contribute critically to immune inflammation by facilitating the differentiation and pathogenic conversion of Th1 and Th17 cells and that various PGs are also involved in different aspects of Th2‐mediated allergic inflammation. Studies have also appeared to indicate that PGs are involved in the modulation of innate lymphoid cells (ILCs), a class of lymphoid cells mirroring T‐cells but not expressing the T‐cell receptor (TCR). Figure 2 summarizes the actions of PGs on different inflammatory responses driven by different subsets of T and ILC cells, which will be discussed in detail below.

### PG signalling in immune inflammation mediated by Th1 and Th17 cells

#### PGE_2_‐EP_2_/EP_4_ receptor signalling in Th1 differentiation

**Figure 3:**
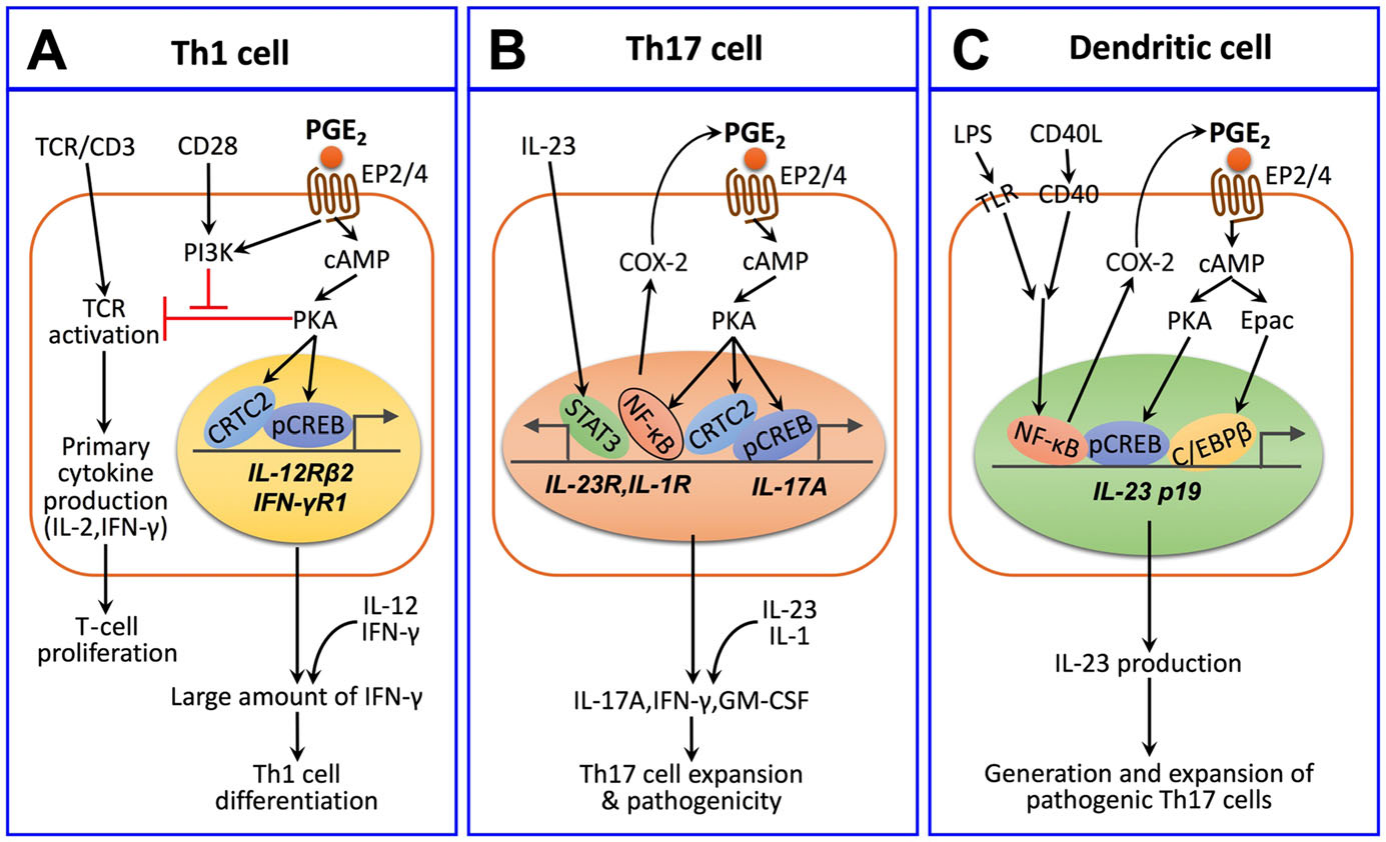
Molecular mechanisms for PGE_2_‐cytokine crosstalk in facilitation of adaptive Th1 and Th17 cell responses. (A) PGE_2_ through EP_2_/EP_4_ receptors activates the cAMP‐PKA‐CREB/CRTC2 pathway that in turn drives the expression of IL‐12β2 and IFN‐γ 1 receptors (R) and amplifies cytokine signalling, leading to facilitation of Th1 cell differentiation. Additionally, activation of PI3K by strengthened CD28 costimulation and that by EP_2_/EP_4_ receptors further supports Th1 cell differentiation by antagonizing cAMP/PKA‐mediated suppression of TCR activation. (B) IL‐23 induces COX‐2 expression and low levels of PGE_2_ production by activated Th17 cells. T‐cell‐intrinsic PGE_2_ then acts back on Th17 cells for the induction of IL‐23 receptor (and possibly also IL‐1 receptor) through the positive feedback IL‐23‐PGE_2_‐EP_2_/EP_4_‐cAMP‐PKA‐STAT3/CREB/NF‐κB‐IL‐23 receptor loop, leading to the generation of pathogenic Th17 cells by synergistic actions with IL‐23 and/or IL‐1. CREB and CRTC2 can also directly drive IL‐17A gene expression by binding to its promoter. (C) Activation of NF‐κB induced by TLR or CD40 engagement on DCs induces the early phase of IL‐23 p19 gene expression. NF‐κB also induces DCs to express COX2 and PGE_2_, which then further amplifies NF‐κB signalling for the IL‐23 p19 expression through the EP_2_/EP_4_‐cAMP‐PKA‐CREB pathways. PGE_2_ can also induce IL‐23 p19 expression through cAMP‐Epac pathway‐activated C/EBPβ.

Differentiation of Th1 cells is driven by two critical cytokines, IL‐12 and IFN‐γ. Upon TCR engagement, T‐cells produce a small amount of IFN‐γ that binds to the IFN‐γ receptor and induces a low level of IL‐12 receptor β2 chain (IL‐12Rβ2), enabling T‐cell responses to IL‐12. The IL‐12–IL‐12 receptor interaction then activates transcription factors T‐bet and STAT4 and amplifies the expression of IL‐12Rβ2 as well as producing a large amount of IFN‐γ for Th1 differentiation (Zhu *et al*., 2010). PGE_2_ has been believed for a long time to act as an immunosuppressant, suppressing Th1 cell development and function in *in vitro* studies (Betz and Fox, 1991; Harris *et al*., 2002). However, recent studies indicate that the inhibitory action of PGE_2_ depends on the extent of TCR stimulation as well as co‐existing cytokines. Substantial *in vitro* and *in vivo* evidence now shows that PGs can act as an immune‐activator under numerous conditions. Indeed, in the presence of enhanced TCR stimulation, PGE_2_ facilitated, rather than inhibited, IL‐12‐primed Th1 cell differentiation, and this was mediated by EP_2_ and EP_4_ receptors through the cAMP‐PKA pathway (Yao *et al*., 2009). Furthermore, PGE_2_ promotes Th1 cell differentiation by induction of the IL‐12Rβ2 and IFN‐γ receptor α chain (IFN‐γ receptor 1), thus amplifying the IL‐12 and IFN‐γ signalling pathways (Yao *et al*., 2013). Mechanistically, PGE_2_‐EP_2_/EP_4_‐cAMP‐PKA signalling activates cAMP‐response element binding protein (CREB) by CREB phosphorylation and its cofactor CREB‐regulated transcription co‐activator 2 (CRTC2) through CRTC2 de‐phosphorylation (Screaton *et al*., 2004), both of which then translocate to the nucleus, bind to IL‐12Rβ2 and IFN‐γ receptor 1 gene loci to initiate their transcription (Yao *et al*., 2013). These results have thus clearly established that the PGE_2_‐EP_2_/EP_4_‐cAMP‐PKA pathway can facilitate Th1 cell development through crosstalk with IL‐12 and IFN‐γ (Figure 3A). In addition to PGE_2_, PGI_2_ appears to similarly promote Th1 differentiation possibly through cAMP‐PKA signalling (Nakajima *et al*., 2010).

#### PGE_2_‐EP_2_/EP_4_ receptor signalling in expansion and pathogenic conversion of Th17 cells

Th17 cells drive chronic immune inflammation through the pro‐inflammatory cytokines they produce, for example, IL‐17A, IL‐17F and IL‐22 (Stockinger and Omenetti, 2017). Mouse Th17 cells are induced from naïve T‐cells by TGF‐β and IL‐6 and then expanded and maturated by IL‐23. Primarily differentiated Th17 cells express relatively lower amounts of IL‐17A and **IL‐23 receptors** and have less pathogenicity. Once these cells are further stimulated by IL‐23, they express high levels of IL‐23 receptor, produce large amounts of IL‐17A and other cytokines (such as IL‐17F, IL‐22 and GM‐CSF) and become more pathogenic (Lee *et al*., 2012). Human Th17 cells can be induced by IL‐23 and IL‐1β either with or without TGF‐β. Unlike Th1 cells, Th17 cell differentiation by TGF‐β and IL‐6 is inhibited by PGE_2_, possibly due to a down‐regulation of TCR signalling and TGF‐β signalling. However, PGE_2_ markedly enhances IL‐23‐stimulated Th17 cell proliferation and the production of IL‐17 and IL‐22, an action also mediated by EP_2_ and EP_4_ receptors through the downstream cAMP‐PKA pathway (Yao *et al*., 2009). Intriguingly, suppression of endogenous cAMP production by a deficiency in the stimulatory Gα subunit of the heterotrimeric G protein on T‐cells down‐regulated Th17 and Th1 cell differentiation (Li *et al*., 2012). Recently, Lee *et al*. examined the molecular mechanisms of PGE_2_‐EP_2_/EP_4_ receptor signalling‐mediated amplification of IL‐23‐induced Th17 expansion in detail and found that this PGE_2_ pathway amplifies IL‐23‐induced IL‐23 receptor expression by not only further activating STAT3 but also activating NF‐κB and CREB through cAMP (Lee *et al*., 2018). Interestingly, they found that IL‐23 induces the expression of COX‐2 in Th17 cells and inhibition of PG synthesis with indomethacin attenuates the IL‐23 action, suggesting that IL‐23 mobilizes endogenous Th17 cell‐intrinsic PGE_2_ system to augment its effects. They further showed that the cAMP pathway not only enhances IL‐23‐induced gene expression but also induces the expression of a variety of genes that are not induced by IL‐23 alone and confers more pathogenic signature to Th17 cells (Lee *et al*., 2018). The involvement of CREB and its co‐activator CRTC2 in promoting Th17 cells were also reported by other groups (Hernandez *et al*., 2015; Wang *et al*., 2017). These studies suggested that the promoting action of PGE_2_ on pathogenic Th17 cells is, at least partly, due to direct binding of CREB and CRTC2 to an IL‐17A promoter and enhancing its gene transcription (Figure 3B).

The PGE_2_‐EP_2_/EP_4_‐cAMP signalling pathway also promotes IL‐23/IL‐1β‐stimulated expansion and activation of human Th17 cells (Chizzolini *et al*., 2008; Boniface *et al*., 2009; Napolitani *et al*., 2009). As seen in mouse T‐cells, PGE_2_ signalling not only directly enhances the induction of IL‐17A gene transcription but also induces receptors for the cytokines involved. Boniface *et al*. observed an up‐regulation of the expression of IL‐23 and IL‐1 receptors in PGE_2_‐stimulated human Th17 cells and proposed that PGE_2_ amplifies IL‐23‐IL‐23 receptor and IL‐1β‐IL‐1 receptor signalling to optimize IL‐17A production. They further reported that of the two EP receptors, EP_2_ is predominantly responsible for Th17 cytokine production, while EP_4_ exerts the additional effects such as inhibiting IL‐10 and IFN‐γ production in Th17 cells (Boniface *et al*., 2009). Consistent with these findings, blockade of transcriptional co‐activators of CREB, CREB binding protein (CBP) and p300, by the bromodomain CBP30 inhibits IL‐17A secretion by human Th17 cells (Hammitzsch *et al*., 2015). Furthermore, a recent study demonstrated that the EP_2_ receptor is overexpressed in T‐cells from MS patients, while its expression in T‐cells in healthy individuals undergoes retinoic acid‐related orphan nuclear hormone receptor C‐dependent silencing and that simulation of EP_2_ receptors in the MS patient T‐cells induces a pathogenic Th17 phenotype characterized by co‐expression of both IL‐17A and IFN‐γ (Kofler *et al*., 2014), thus providing clinical relevance of the finding on PGE_2_‐EP_2_ receptor signalling in mouse Th17 cells. Taken together, these studies clearly indicate the important roles for PGE_2_ in the development of pro‐inflammatory Th17 cells and, as described later, the pathogenesis of chronic inflammation in the mouse and human (Figure 3B).

The discrepancy between previous observations on PGE_2_ suppression of T‐cell activation, proliferation and cytokine production (Harris *et al*., 2002) and recent findings about PGE_2_ facilitation of Th1 and Th17 cell responses (Sheibanie *et al*., 2007a; Chizzolini *et al*., 2008; Boniface *et al*., 2009; Yao *et al*., 2009, 2013; Chen *et al*., 2010; Lee *et al*., 2018) may be caused by multiple factors. One is that different concentrations of PGE_2_ were used in the experiments. In most previous studies with *in vitro* cell cultures, PGE_2_ was used at micromolar levels, but actual concentrations of PGE_2_ *in vivo*, especially in humans, are usually at nanomolar levels, examples being those in cerebral spinal fluid from MS patients (Mattsson *et al*., 2009) and in joint fluid from RA patients (Hishinuma *et al*., 1999). PGE_2_ was found at similar levels (ng·g^−1^ wet weight of tissue) in the rectal mucosa from UC patients (Sharon *et al*., 1978) and in colonic mucosa from rat with dextran sulfate sodium (DSS)‐induced colitis (Yamashita, 1993). TCR‐dependent T‐cell activation and function was inhibited by high rather than low (~nM) concentrations of PGE_2_. In contrast, the low, nanomolar concentrations of PGE_2_ facilitated Th1 and Th17 cell responses in both *in vitro* and *in vivo* assays (Yao *et al*., 2009, 2013; Lee *et al*., 2018). Another factor may be the different T‐cell stimulation conditions, for example, the strength of TCR‐costimulation and the cytokine milieu present during T‐cell differentiation and expansion (Yao *et al*., 2013; Lee *et al*., 2018). Altogether, PGE_2_ can have both pro‐ and anti‐inflammatory effects depending on its local concentrations, the disease settings and also the timing of its action.

#### PGE_2_‐EP_2_/EP_4_ receptor signalling in migration, maturation and IL‐23 production of DCs

The involvement of PGs in acquired immunity is not limited to its regulation of T‐cells but is also seen in APCs. Kabashima *et al*. found that, in response to antigen uptake, the migration of Langerhans cells to regional lymph nodes, the expression of costimulatory molecules and their ability to stimulate T‐cells were impaired in EP_4_‐deficient mice, suggesting a critical role for PGE_2_‐EP_4_ receptor signalling in DC maturation and migration after antigen uptake (Kabashima *et al*., 2003b). PGE_2_ was also reported to up‐regulate **OX40 ligand** (OX40L) expression on human monocyte‐derived DCs, which in turn enhanced the **OX40**‐OX40L costimulation to enhance human antigen‐specific T‐cell proliferation in *in vitro* T‐cell‐DC co‐cultures (Krause *et al*., 2009). PGE_2_ is further involved in IL‐23 production by activated DCs. Ganea's group showed that various toll‐like receptor (TLR) ligands such as LPS, poly‐I‐C, CpG and proteoglycan induce the expression of IL‐23 p19 subunit in DCs and that PGE_2_ potently enhances this action *via* EP_2_ and EP_4_ receptors *in vitro* (Sheibanie *et al*., 2004; Khayrullina *et al*., 2008). In the animal model of collagen‐induced arthritis, administration of PGE_2_ analogue, misoprostol, exacerbated joint inflammation, and this was associated with increased mRNA levels of IL‐23 p19 and other pro‐inflammatory cytokines such as IL‐17, IL‐6 and IL‐1β (Sheibanie *et al*., 2007a). This PGE_2_ action is exerted by an interaction between NF‐κB activated by the TLR pathway and CREB and C/EBP‐β activated by the PGE_2_‐EP_4_‐cAMP‐PKA and Epac pathways respectively (Kocieda *et al*., 2012). The PGE_2_‐mediated enhancement of IL‐23 production was also seen in DCs stimulated with anti‐CD40 antibody and, interestingly, treatment with indomethacin or an EP_4_ antagonist almost completely suppressed IL‐23 production *in vitro* (Yao *et al*., 2009), suggesting that DC‐intrinsic PGE_2_ amplifies IL‐23 production through a positive feedback loop. A further study by Ma *et al*. showed that anti‐CD40 antibody stimulation induced two phases of IL‐23 gene expression, the first with a peak at 1 h and the second lasting up to 36 h, while LPS or TNF‐α induced only the early response of IL‐23 gene expression. PGE_2_ or an EP_4_ agonist but not an EP_2_ agonist amplified both phases of the anti‐CD40 response by tens of folds (Ma *et al*., 2016). Mechanistic analysis revealed that the early phase was mediated by canonical NF‐κB signalling and the late phase by non‐canonical NF‐κB signalling and that PGE_2_‐EP_4_ signalling exhibited a synergistic action with both signalling pathways (Figure 3C). One pathological implication of the IL‐23 induction by this PGE_2_ signalling is in cancer. The IL‐23 to IL‐17 cascade through the IL‐23 receptor is also implicated in various human cancers including those of the colon, ovary, lung, breast, stomach, skin, liver and head and neck (Wang and Karin, 2015). Qian *et al*. found that mice bearing 4T1 breast tumour cells have an increased number of Th17 cells not only in tumour tissues but also in the spleen and peripheral blood and that this is due to PGE_2_ secreted from the tumour, which induces IL‐23 in DCs through EP_2_/EP_4_ receptors and a CREB‐dependent manner (Qian *et al*., 2013). In addition, Kabashima *et al*. reported that the engagement of TP receptors in T‐cells with TXA_2_ produced by DCs resulted in impaired DC‐T‐cell adhesion and inhibited DC‐dependent T‐cell proliferation *in vitro*, while TP‐deficient or TP antagonist‐treated mice had enhanced acquired immune response to foreign antigens *in vivo* (Kabashima *et al*., 2003a). A further study indicates that TXA_2_ tonically suppresses the interaction of weak CD4^+^ T‐cells and DCs through TP receptors (Moalli *et al*., 2014).

#### PGE_2_‐EP_2_/EP_4_ signalling in Th1/Th17‐mediated immune inflammation; animal models and human relevance

Given the PGE_2_‐EP_2_/EP_4_‐mediated facilitation of differentiation of Th1 cells, expansion and pathogenic conversion of Th17 cells and IL‐23 production by DCs, several studies have examined the significance of this signalling in Th1‐ and Th17‐driven immune inflammation in various animal models of autoimmune diseases such as MS (Kihara *et al*., 2009; Yao *et al*., 2009; Esaki *et al*., 2010; Hernandez *et al*., 2015), IBD (Sheibanie *et al*., 2007b; Wang *et al*., 2017), arthritis (Chen *et al*., 2010; Monk *et al*., 2014), allergic lung inflammation (Li *et al*., 2011) and atherosclerosis (Kotla *et al*., 2013) and their relevance to human autoimmune diseases. For example, blockade of PGE_2_‐EP_4_ receptor signalling by pharmacological (e.g. COX inhibitors and EP_4_ antagonists) or genetic approaches (e.g. KOs of mPGES1 or EP_4_ genes) suppressed myelin oligodendrocyte glycoprotein peptide‐induced experimental autoimmune encephalomyelitis (EAE) and reduced Th1 and Th17 cell development, and these suppressing effects were further enhanced by co‐inhibition of EP_2_ receptors (Kihara *et al*., 2009; Yao *et al*., 2009; Esaki *et al*., 2010). Pharmacological antagonism of EP_4_ receptors also attenuated collagen‐induced arthritis progression with concomitant inhibition of the production of IFN‐γ and IL‐17A (Sheibanie *et al*., 2007a; Chen *et al*., 2010). While conditional deletion of EP_4_ receptors in T‐cells ameliorated T‐cell‐mediated chronic colitis accompanied by reduction in Th1 accumulation in the colon (Yao *et al*., 2013), the administration of PGE_2_ or **misoprostol**, an EP_3_/EP_4_ receptor agonist, augmented 2,4,6‐trinitrobenzene sulfonic acid‐induced colitis with an increase in IL‐17 and IL‐23 levels in inflamed colon (Sheibanie *et al*., 2007b). Recently, Lee *et al*. showed that T‐cell‐specific deletion of EP_2_ and EP_4_ receptors in combination suppressed psoriasis‐like skin inflammation induced by subcutaneous injection of IL‐23 or imiquimod with concomitant suppression of IL‐17^+^ and IL‐17^+^IFN‐γ^+^ T‐cell accumulation in the lesion, suggesting the critical role of T‐cell‐intrinsic EP_2_/EP_4_ receptor signalling in the development of immune inflammation (Lee *et al*., 2018). Moreover, deletion of Gαs or CREB in T‐cells similarly reduced Th17‐induced chronic colitis and EAE (Li *et al*., 2012; Wang *et al*., 2017). Although PGE_2_ essentially protects against acute epithelial injury (Kabashima *et al*., 2002; Duffin *et al*., 2016), these studies together suggested the detrimental action of PGE_2_ signalling in Th17 cells in the gut. Similar to PGE_2_, activation of PGI_2_‐IP receptor signalling has also been reported to foster Th17 cell responses and exacerbate chronic inflammation mediated by this type of cell in EAE and enhance activation of Th17 cells from systemic sclerosis patients (Truchetet *et al*., 2012; Zhou *et al*., 2012). Therefore, blockade of PGE_2_ or PGI_2_ production by inhibiting COX, mPGES1 or their receptors by small molecular inhibitors would be an effective treatment in the progression of autoimmune diseases.

The clinical relevance of these findings to human autoimmune diseases is indicated by experimental findings that patients with autoimmune inflammatory diseases like IBD and MS have elevated levels of PGE_2_ in their serum and the site of inflammation, for example, the cerebral spinal fluid for MS (Mattsson *et al*., 2009; Prüss *et al*., 2013), and genetic findings from recent GWAS studies, which have identified *Ptger4* (EP_4_ receptor) as a susceptible locus in a number of autoimmune diseases including IBD (Glas *et al*., 2012), MS (International Multiple Sclerosis Genetics Consortium *et al*., 2011), ankylosing spondylitis (AS) and allergy (Hinds *et al*., 2013; Parkes *et al*., 2013). Numerous GWAS studies have suggested that polymorphisms in the 5p13.1 regulatory regions near *PTGER4* (e.g. rs9292777, rs7725052, rs77145747, rs6896969 and rs4613763) were significantly associated with *PTGER4* gene expression and the susceptibility of MS (De Jager *et al*., 2009; International Multiple Sclerosis Genetics Consortium *et al*., 2011; Matesanz *et al*., 2012). In human IBD, gene polymorphisms in *PTGER4* loci (e.g. rs4613763, rs16869977, rs10512739, rs6880934 and rs9292777) were similarly associated with the susceptibility of Crohn's disease (CD) (Libioulle *et al*., 2007; Barrett *et al*., 2008; Kenny *et al*., 2012). Further studies indicated that rs7720838 and rs4495224 were also associated with susceptibility to CD and contributed to increased *PTGER4* gene expression by enhancing the binding of NF‐κB and XBP1 (Glas *et al*., 2012) and that the disease behaviour in CD patients was enhanced if mutant alleles in both rs7720838 and NOD2 were present (Prager *et al*., 2014). PGs (e.g. PGE_2_ and PGI_2_) also promote chronic joint inflammation, and NSAIDs are the first line medications for treating arthritis. Polymorphisms (rs10440635 and rs76523431) in *PTGER4* loci were associated with increased *PTGER4* gene expression in synovial biopsy samples from patients with spondyloarthritis but also the susceptibility and severity of AS (Evans *et al*., 2011; Chai *et al*., 2013). Interestingly, polymorphisms in PTGER4 gene (rs12186979 and rs13354346) were related to susceptibility of AS in Europeans and East Asians respectively (International Genetics of Ankylosing Spondylitis Consortium *et al*., 2013). In addition, rs76523431 in *PTGER4* was also suggested as a risk factor for RA (Rodriguez‐Rodriguez *et al*., 2015). *PTGER4* gene polymorphisms (rs7720838 and rs1494558) were associated with allergy and asthma (Kurz *et al*., 2006; Hinds *et al*., 2013), while rs4613763 was suggested as a psoriasis susceptible locus (Tsoi *et al*., 2015). Furthermore, epigenetic changes in the *PTGER4* gene enhancer in Th17 cells were also found to be associated with human autoimmune diseases such as MS, CD and allergy (Farh *et al*., 2015). Consistent with these GWAS findings, the expression of PGE_2_ signalling pathway genes (including both PGE_2_ synthases and receptors) was found to be positively correlated with IL‐23/Th17 signature genes as well as disease severity in biopsy samples of human inflamed tissues with various chronic inflammatory conditions such as MS, CD, psoriasis and atopic dermatitis (Lee *et al*., 2018; Robb *et al*., 2018; and unpublished observations). Collectively, these genetic and epigenetic studies revealed conserved associations of variants in the PGE_2_‐EP_4_ signalling pathway with various IL‐23/Th17‐dependent human chronic autoimmune diseases.

### PGE_2_‐EP_4_ receptor signalling in the generation of IL‐22‐producing Th22 cells and Th22‐mediated inflammation

IL‐22 that is produced by Th17 cells and other activated T‐cells such as Th22 cells is involved in inflammation of the skin, gut, liver, lung as well as infections and tissue remodelling (Sabat *et al*., 2014). Robb *et al*. recently reported that PGE_2_ promotes IL‐22 production from T‐cells under Th22‐skewing conditions with IL‐23 or IL‐6 or both, and this effect was mediated by the EP_2_/EP_4_‐cAMP‐PKA pathway through induction of the aryl hydrocarbon receptor. Consistently, T‐cell‐specific EP_4_ receptor deficiency as well as COX inhibition reduced hapten (e.g. dinitro‐fluorobenzene)‐induced generation of IL‐22^+^ T‐cells *in vivo* and attenuated chronic allergic contact dermatitis induced by repeated oxazolone challenge (Robb *et al*., 2018). Interestingly, genes related to PGE_2_ and IL‐22 pathways were coordinately up‐regulated in lesional skin from human atopic dermatitis, and their expression in inflamed skin was also down‐regulated after the administration of corticosteroid or UVB treatments (Robb *et al*., 2018). These results suggest a crucial role for PGE_2_‐IL‐6/IL‐23 crosstalk in the generation of IL‐22^+^ T‐cells and the promotion of T‐cell‐mediated chronic inflammatory skin disease.

### PG signalling in Th2 cell‐mediated allergic inflammation

Unlike Th1 and Th17 cell differentiation, DCs do not produce IL‐4, the key cytokine for Th2 differentiation, and the differentiation of Th2 cells is induced by direct and indirect interaction of T‐cells with DCs, basophils, ILCs, epithelial cells and maturing Th2 cells themselves and factors released from these cells, such as the epithelial cell‐produced cytokines, IL‐25, IL‐33 and TSLP, and IL‐4 produced by maturing Th2 cells and basophils (Walker and McKenzie, 2017). Th2 cells not only provide protective type 2 immune responses against parasites but also underpin the chronic, allergic inflammatory diseases such as atopic dermatitis and asthma. In contrast to PGE_2_‐facilitated Th1 differentiation and Th17 expansion, most PGs appear to suppress Th2 differentiation. Nakajima *et al*. found that IP receptor stimulation of IP concentration‐dependently facilitates Th1 differentiation under the Th1 skewing conditions but suppresses Th2 differentiation of BALB/c CD4^+^ T‐cells under the Th2‐skewing condition. This IP receptor‐mediated pathway appears to suppress Th2 cell differentiation *in vivo* (Nakajima *et al*., 2010). Indeed, mice deficient in IP receptors exhibited significantly higher serum IgE levels compared to wild type mice and showed significantly enhanced allergic inflammation of the lung in an ovalbumin (OVA)‐induced allergic asthma model (Nagao *et al*., 2003). Zhou *et al*. confirmed these phenotypes of IP‐deficient mice and further revealed that these phenotypes caused by IP‐deficiency are not dependent on STAT6 (Zhou *et al*., 2016b). Similar to IP receptor‐deficient mice, EP_2_ receptor‐deficient mice also show an exaggerated OVA‐induced airway inflammation accompanied by increased IL‐13 production, with higher serum IgE levels, and the administration of misoprostol during the sensitization process suppressed the production of IgE and attenuated the inflammatory response (Zasłona *et al*., 2014). The same phenotype, that is, exaggerated allergic inflammation with higher IgE levels, was also found in mice deficient in either COX‐1 or COX‐2 subjected to the OVA model. These results suggest that the PGE_2_‐EP_2_ receptor axis and the PGI_2_‐IP receptor axis function as an endogenous brake on allergen sensitization of T‐cells, although the detailed mechanism remains unknown.

In addition to these analyses on the role of PGs in allergen sensitization, several studies have also revealed the involvement of PGs in the effector phase of Th2 cell‐driven allergic inflammation. The best analysed PG in this phase is PGD_2_, which is abundantly produced by mast cells activated by allergens. PGD_2_ acts on DP_1_ and DP_2_ receptors, and both PGD receptors regulate allergic inflammation positively. Th2 cells highly express DP_2_ receptors, and PGD_2_ binding to DP_2_ receptors on Th2 cells stimulates their migration to inflamed sites and enhances type 2 cytokine production (Hirai *et al*., 2001; He *et al*., 2010). Pharmacological inhibition of DP_2_ receptors using small molecule inhibitors has been tested in randomized clinical trials for their efficacy to treat asthma and exhibited significant improvements in lung function and inflammation (Walker and McKenzie, 2017). It is unclear whether they only targeted Th2 cells, because eosinophils, mast cells and type 2 ILCs (ILC2s) also express DP_2_ receptors. Screening studies using mice deficient in each PG receptor suggest an important role of another PGD receptor, DP_1_, in allergic asthma (Matsuoka *et al*., 2000). A deficiency in DP_1_ receptors did not affect serum IgE levels in OVA‐challenged mice but reduced the production of antigen‐specific Th2 cytokines, the accumulation of T‐cells and eosinophils, epithelial mucus secretion and airway hypersensitivity in the sensitized lung (Matsuoka *et al*., 2000). This phenotype in DP_1_‐KO mice was mimicked by administration of a DP_1_‐selective antagonist, S‐5751, to sensitized wild mice (Arimura *et al*., 2001). Interestingly, the expression of DP_1_ receptors in the lung was increased after OVA challenge, and the immune‐reactivity was localized in the airway epithelial cells, suggesting that PGD_2_ may activate epithelial cells by binding to DP_1_ receptor (Arimura *et al*., 2001). In contrast to these actions of PGD_2_, PGE_2_ appears to exert suppressive actions also in the effector phase of type 2 allergic inflammation through various receptors. Kunikata *et al*. found that, compared with WT mice, EP_3_‐deficient mice had increased type 2 cytokine production and T‐cell infiltration in the lung and augmented airway inflammation induced by OVA (Kunikata *et al*., 2005). They further found that OVA challenge induced the expression of a variety of genes related to allergic inflammation and tissue remodelling including the chemokine thymus and activation‐regulated chemokine (TARC, also known as **CCL17**) and eotaxin, in the lung, and the EP_3_ agonist treatment suppressed this induction and the above asthmatic symptoms. Intriguingly, EP_3_ receptors and CCL17 are both apparently expressed in the airway epithelium and agonist administration suppressed the expression of CCL17 (Kunikata *et al*., 2005), suggesting that PGE_2_‐EP_3_ receptor signalling inhibits allergic inflammation by suppressing the allergen‐induced gene expression in the epithelium. Because DP_1_ receptor is also expressed in the airway epithelium and exerts opposite actions to EP_3_, and EP_3_ receptors and DP_1_ receptors are coupled oppositely to adenylate cyclase *via* Gαi and Gαs, respectively, these results suggest that the airway epithelium is the site of action of these PGs. In addition to Th2 cells, T‐cells expressing IL‐9 named Th9 cells contribute to allergic inflammation by promoting mast cell expansion and IL‐13 production. Li *et al*. examined the role of PGs in Th9 generation by subjecting COX‐1‐ and COX‐2‐deficient mice to the OVA model and found enhanced generation of Th9 cells in the absence of COX‐2. They then found that PGD_2_ and PGE_2_ suppressed *in vitro* Th9 differentiation induced by TGF‐β and IL‐4 through the cAMP pathway, which was due to a down‐regulation of IL‐17RB that was responsible for the expansion of Th9 induced by IL‐25 endogenously produced in T‐cells during the differentiation of Th9 cells (Li *et al*., 2013). These studies collectively suggest that PGs have diverse effects on Th2‐cell mediated type 2 immune responses and allergic inflammation, which is likely determined by the balance of different PGs during antigen sensitization and challenge. In addition to the above studies focused on allergic inflammation, Birrell *et al*. examined EP receptors in PGE2‐mediated modulation of anti‐inflammatory and bronchodilator activities in the lung and found that EP4 agonists can control airway inflammation in various models induced by endotoxin, OVA or cigarette smoke (Birrell *et al*., 2015).

### PG signalling in inflammation mediated by innate lymphoid cells

ILCs are recently identified new types of innate immune cells with transcriptional, functional and phenotypic similarities to Th subsets but without lymphocyte lineage markers and antigen receptors. ILCs therefore respond not to antigens but to environmental factors such as cytokines and lipid mediators. Similar to Th subsets, ILCs are classified into three subsets, ILC1, ILC2 and ILC3, according to their cytokine production and master transcription factors; ILC1s produce IFN‐γ in response to IL‐12, ILC2s produce type 2 cytokines such as IL‐5 and IL‐13 in response to epithelial cytokines (alarmins), IL‐33, IL‐25 and TSLP, and ILC3s produce IL‐22 and/or IL‐17 in response to IL‐23 and IL‐1β. (Klose and Artis, 2016). As indicated from the above properties, ILC2s mediate type 2 immune responses and allergic diseases like Th2 cells in response to alarmins, various cytokines and lipid mediators. The most well‐studied PG affecting ILC2 responses is PGD_2_. Like Th2 cells, human ILC2 cells highly express the PGD receptor DP_2_ (Mjösberg *et al*., 2011). The stimulation of DP_2_ receptors by PGD_2_ enhanced ILC2 migration and induced the expression of not only genes for type 2 cytokines such as IL‐4, IL‐5 and IL‐13 but also genes for IL‐33 and Il‐25 receptor subunits, **ST2** and IL‐17B receptors, respectively, thus amplifying IL‐33 and IL‐25 signalling pathways for the production of type 2 cytokines and promoting allergic inflammation in skin and lung (Chang *et al*., 2014; Xue *et al*., 2014). Eastman *et al*. reported that ILC2 cells were specifically recruited to nasal mucosa in patients with aspirin‐exacerbated respiratory disease during aspirin challenge, with a paradoxical increase in urinary PGD_2_ metabolite, 11β‐PGF_2_ (Eastman *et al*., 2017). This study indicates that PGD_2_ may have critical roles in recruiting and activating ILC2s through DP_2_ receptors in aspirin‐exacerbated respiratory disease. PGD_2_ similarly induces ILC2 chemotaxis in a DP_2_ receptor‐dependent manner in mice. Wild type mice infected with helminth exhibited pulmonary ILC2 accumulation and type 2 inflammation, which was significantly reduced by a deficiency in DP_2_ receptors and the administration of a DP_2_ antagonist (Wojno *et al*., 2015), demonstrating that the PGD_2_‐DP_2_ axis is indeed involved in ILC2 recruitment *in vivo*. In contrast to PGD_2_, PGE_2_ suppresses the up‐regulation of GATA3 and CD25 (i.e. IL‐2 receptor‐α) induced by IL‐33/IL‐25/TSLP in human ILC2 cells and inhibits ILC2 proliferation and expression of type 2 cytokines such as IL‐5 and IL‐13 through EP_2_ and EP_4_ receptors (Maric *et al*., 2018). In addition, PGI_2_ also inhibited IL‐33‐stimulated ILC2 activation and type 2 cytokine production *in vitro*. The administration of cicaprost, a synthetic IP agonist, suppressed the ILC2 response and lung inflammation induced by a fungal aeroallergen while a deficiency in IP receptors augmented this ILC2‐dependent *in vivo* allergic immune response, suggesting a critical role for endogenous PGI_2_‐IP receptor signalling in the control of ILC2‐mediated allergic inflammation (Zhou *et al*., 2016a).

As for the role of PGs in ILC3, Duffin *et al*. recently reported that PGE_2_ promoted IL‐23‐induced ILC3 activation and IL‐22 production *in vitro*, and this was mediated by EP_2_/EP_4_‐cAMP‐PKA signalling (Duffin *et al*., 2016). Given that IL‐22‐producing ILC3 cells play important roles in mucosal homeostasis and control of acute epithelial damage (Klose and Artis, 2016), they examined the significance of this pathway in LPS‐induced systemic inflammation. They found that inhibiting COX with indomethacin markedly exacerbated LPS‐induced systemic inflammation, which was caused by enhanced gut bacterial translocation due to reduced number of ILC3 cells and IL‐22 production in the intestine and that all of these phenotypes could be prevented by administration of an EP_4_ selective agonist or exogenous IL‐22. They further showed that EP_4_ deletion in ILCs led to a reduction of IL‐22 production and augmented systemic inflammation, as exemplified by enhanced TNF‐α production. Moreover, PGE_2_ also enhanced IL‐22 production from IL‐23/IL‐1β‐stimulated human ILC3s. These results together suggest that PGE_2_‐EP_4_ receptor signalling directly acts on ILC3s to potentiate their homeostasis and function in the intestine, promoting its barrier function to prevent systemic and intestinal inflammation (Duffin *et al*., 2016). In addition to this cytoprotective effect, IL‐22‐producing ILC3 cells sustain colon cancer in a colitis‐associated cancer model of 129SvEv.Rag^−/−^ mice treated with *Helicobacter hepaticus* and azoxymethane (AOM) (Kirchberger *et al*., 2013), and some ILC3s also produce pro‐inflammatory cytokines (e.g. IL‐17A, IFN‐γ and GM‐CSF) in response to IL‐23 and other cytokines, such as IL‐1β and IL‐12 (Klose and Artis, 2016), which contribute to intestinal inflammation (Buonocore *et al*., 2010). Whether PGE_2_ or other PGs can also promote such mucosal inflammation through stimulating the pro‐inflammatory ILC3s similarly to pathogenic Th17 cells by interacting with IL‐23 and/or IL‐1β is not known.

### PG signalling in the regulation of B cells responses

Previously, PGE_2_ was shown to be involved in the modulation of immunoglobulin class switching to IgG1 and IgE synthesis *in vitro* through EP_2_/EP_4_‐cAMP signalling (Harris *et al*., 2002). Consistent with these findings, Gao *et al*. found that PGE_2_ enhances IL‐4‐induced STAT6 activation in an EP_2_‐dependent manner to prompt IgE switching and that in the OVA‐induced asthma model, mice with a deficiency in EP_2_ receptors exhibited markedly attenuated IgE antibody responses and airway inflammation (Gao *et al*., 2016). Furthermore, also consistent with the earlier findings on PGE_2_‐mediated inhibition of B cells activation (Roper *et al*., 1994), Murin *et al*. found that PGE_2_ suppresses B cell receptor‐mediated proliferation of B cells lymphomas through EP_4_ receptors and that gene knockdown of *Ptger4* (encoding EP_4_ receptor) in B cells lymphoma markedly accelerates tumour spread in mice, while *PTGER4* overexpression yields significant protection (Murn *et al*., 2008). Because the expression of EP_4_ receptors is down‐regulated in the lymphoma, the authors suggested a tumour‐suppressor role for EP_4_ receptors. Prijatelj *et al*. suggested that PGE_2_‐EP_4_ receptor signalling‐mediated elevation of cAMP and inactivation of NF‐κB by EP_4_ may be involved in the inhibition of B cell proliferation (Prijatelj *et al*., 2012). In the germinal centre (GC), B cells are selected through interaction with follicular DCs bearing immune complexes in an antigen affinity‐dependent manner in the so‐called affinity maturation process, and IL‐21 produced by follicular helper T‐cells stimulates this process. Magari *et al*. constructed a co‐culture of FL‐YB follicular DCs and B cells and found that FL‐YB cells produced PGE_2_, which, combined with IL‐21, acted on EP_4_ receptors and induced B cell death by up‐regulation of pro‐apoptotic proteins Bim and Foxo1 (Magari *et al*., 2011). Since treatment with indomethacin and an EP_4_ antagonist ONO‐AE3‐208 significantly decreased the number of apoptotic GC B cell, these authors indicated that the IL‐21‐PGE_2_ crosstalk physiologically regulates β cell death in GC B cell selection. In addition to these actions of PGs on B cell fate in the periphery, in mice deficient in COX‐1 early B cell development from pro‐B to pre‐B stage was arrested, which caused a systematic reduction in total B cells (Yang *et al*., 2014). Mechanistic studies revealed that COX‐1‐derived TXA_2_ regulated JAK3/STAT5 signalling through binding to TP receptors. Treatment of COX‐1‐deficient mice with a TP agonist restored the defective B cell development and JAK3/STAT5 signalling activity (Yang *et al*., 2014).

## PG‐cytokine crosstalk in myeloid and stromal cell‐mediated chronic inflammation

Chronic inflammation is characterized by the persistent infiltration of mononuclear cells including macrophages, lymphocytes and plasma cells in most cases and, in some cases, polymorphonuclear leukocytes, and consequent tissue destruction largely by the products of these infiltrating inflammatory cells (Kumar *et al*., 2018). Tissue remodelling such as angiogenesis and fibrosis is also simultaneously seen in chronic inflammation. Substantial evidence has now accumulated indicating that PGs contribute to these processes.

### PG‐cytokine crosstalk in macrophages

**Figure 4:**
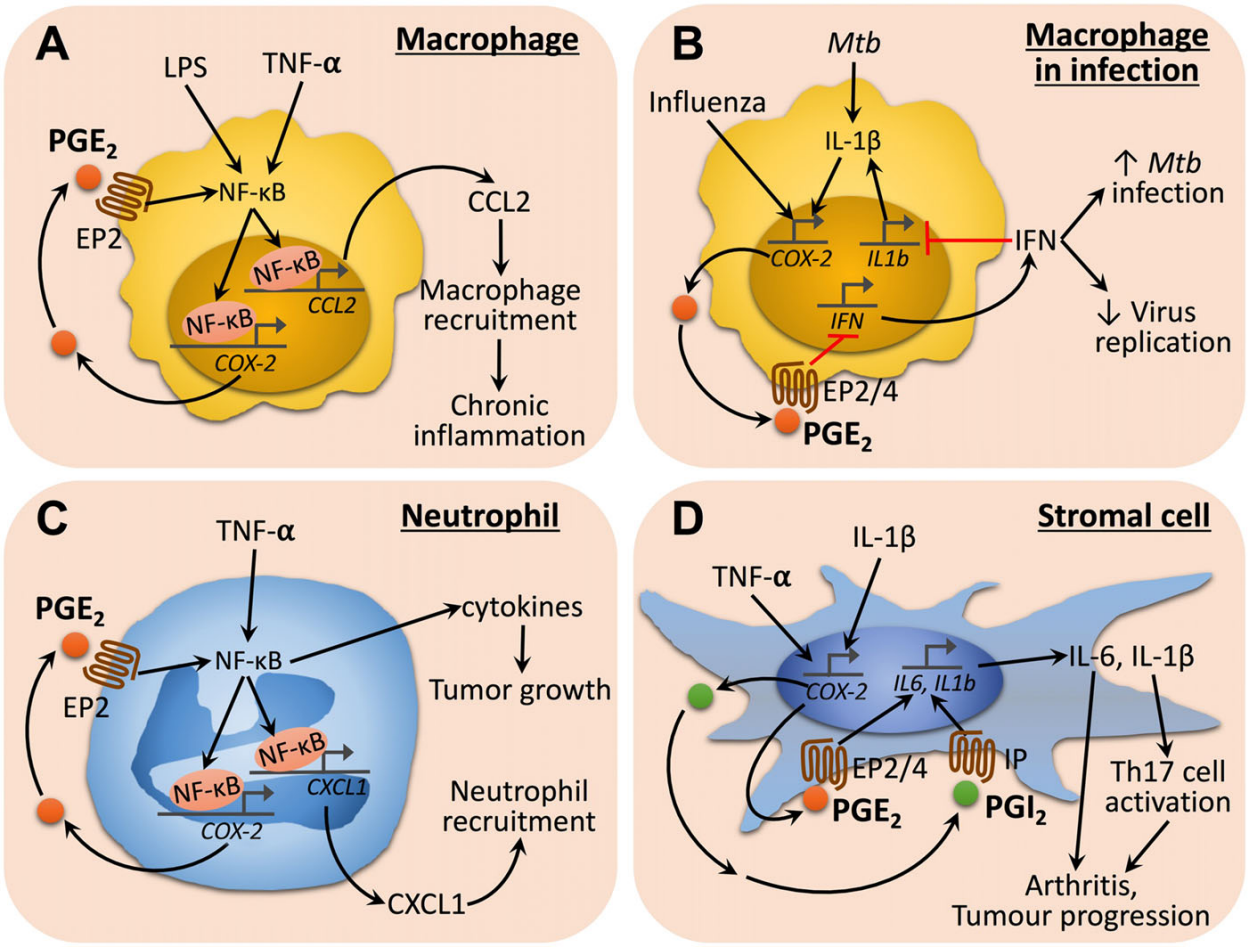
Molecular mechanisms for PG‐cytokine crosstalk in the amplification of innate immune cell responses. (A) TNF‐α and LPS activate NF‐κB in macrophages to express COX‐2 and PGE_2_, which acts back on macrophages *via* EP_2_ receptors to further amplify NF‐κB signalling for the induction and stabilization of CCL2 mRNA, recruiting macrophages to inflamed sites and promoting chronic inflammation. (B) *Mycobacterium tuberculosis* (*Mtb*) infection induces IL‐1 production that in turn drives PGE_2_ production by macrophages. PGE_2_ then suppresses macrophage production of type 1 IFNs *via* EP_2_/EP_4_ receptors, leading to an exacerbation of *Mtb* infection. Similarly, influenza A virus infection also activates macrophage production of PGE_2_, which then suppresses type 1 IFNs to control virus replication. (C) TNF‐α activates NF‐κB in neutrophils to express COX‐2 and PGE_2_, which acts back on neutrophils *via* EP_2_ receptors to amplify NF‐κB signalling for induction of CXCL1 and inflammatory cytokines, leading to the recruitment of neutrophils to chronic inflammation sites. TNF‐α‐activated NF‐κB also induces inflammatory cytokines that promote tumour growth. (D) TNF‐α and IL‐1β activate NF‐κB in stromal cells (e.g. fibroblasts) to express COX‐2 and produce PGs (e.g. PGE_2_ and PGI_2_). PGE_2_ and PGI_2_ act back on stromal cells through EP_2_/EP_4_ and IP receptors, respectively, to induce IL‐6 (likely also IL‐1β) production, supporting activation of residential Th17 cells and leading to chronic inflammation such as RA and tumour progression.

The macrophage is one of the main cell sources for PGs in inflammation and the PGs produced act back on macrophages and amplify their function. For example, while LPS induced the expression of COX‐2, IL‐1β and IL‐6 in cultured macrophages, this induction was attenuated by celecoxib, a COX‐2 inhibitor, or RQ‐00015986, an EP_4_ antagonist, suggesting a positive feedback loop for LPS‐activated endogenous PGE_2_‐EP_4_ receptor signalling in macrophages (Oshima *et al*., 2011). This PG‐mediated positive feedback loop has been shown in various models of chronic inflammation. Intracranial aneurysm (IA) is chronic inflammation of the cerebral artery histologically characterized by degenerative changes in the arterial walls and inflammatory cell infiltration consisting mainly of macrophages (Chyatte *et al*., 1999). Aoki *et al*. identified the PGE_2_‐EP_2_‐NF‐κB signalling cascade in macrophages infiltrating the arterial wall as a factor sustaining the pathogenesis of IA and making the inflammation chronic (Aoki *et al*., 2017). They found that PGE_2_ activates NF‐κB synergistically with TNF‐α *via* EP_2_ receptors in macrophages *in vitro* to induce pro‐inflammatory genes including COX‐2 and the macrophage chemokine **CCL2** (also called MCP‐1) and that CCL2 mRNA is also stabilized by this pathway. Consistent with these *in vitro* findings, mice with macrophage‐specific EP_2_ receptor deletion or with transgenic expression of an IκB mutant that restricts NF‐κB activation showed reduced IA incidence with fewer infiltrated macrophages (Aoki *et al*., 2017). Similarly, administration of an EP_2_ antagonist in a rat model of IA reduced macrophage infiltration and suppressed IA formation and progression (Aoki *et al*., 2011, 2017). The authors suggest that PGE_2_‐EP_2_ receptor signalling not only amplifies inflammation by making a positive feedback loop involving NF‐κB and COX‐2 but also amplifies macrophage recruitment by increasing and sustaining CCL2 expression at the inflamed site by this loop, contributing to the pathogenesis of IA (Figure 4A) (Aoki *et al*., 2017). Indeed, infiltration of macrophage‐expressing COX‐2 and EP_2_ receptors was found in clinical samples of human IA (Aoki *et al*., 2017). However, Kumei *et al*., using a diet‐induced nonalcoholic steatohepatitis model, found that deficiency in IP receptors accelerates disease progression by augmenting histological derangements including cell infiltration accompanied by increased expression of CCL‐2 (also called MCP‐1) and TNF‐α. Such disease progression in WT mice is significantly suppressed by administration of an IP agonist, **beraprost** (Kumei *et al*., 2018). These results indicate that PGI_2_‐IP receptor signalling down‐regulates CCL‐2 and TNF‐α and suppresses inflammation, an opposite action to PGE_2_‐EP_2_ receptor signalling described above and suggest that similar PG signalling exert different actions in different context. Similarly, TXA_2_ can also promote the expression of CCL‐2 and its receptor CCR2 in macrophages. In the carbon tetrachloride‐induced liver injury and repair model, a deficiency in TXA_2_‐TP receptor signalling reduced the accumulation of hepatic CD11b^+^F4/80^+^ macrophages and hepatic expression of CCL‐2, CCR2, IL‐6, TNF‐α and hepatocyte growth factor, leading to impaired liver regeneration (Minamino *et al*., 2012).

Other examples of cytokine‐PG interactions in macrophages have been reported in inflammation associated with bacterial and viral infection (Figure 4B). Mayer‐Barber *et al*. found that *Mycobacterium tuberculosis* infection induced IL‐1α and IL‐1β production, which in turn induced PGE_2_ production by macrophages and enhanced their antimicrobial activity (Mayer‐Barber *et al*., 2014). Type 1 IFNs are known to antagonize the IL‐1 receptor pathway during mycobacterium infection and subvert anti‐tuberculosis host defence. They found that the IL‐1 receptor‐PGE_2_ pathway inhibits type 1 IFN synthesis in macrophages and counter‐regulates their actions. Therefore, blocking IL‐1 receptors or COX‐2 signalling or enhancing IFN signalling caused uncontrolled *M. tuberculosis* infection, leading to mortality and necrotic lung inflammation, but this could be prevented by administration of exogenous PGE_2_ (Mayer‐Barber *et al*., 2014). This study provided a novel mechanism for how the interaction between IL‐1 receptors, PGE_2_ and IFN in macrophages determines outcomes of bacterial infection and suggests potential therapeutic strategies for tuberculosis by targeting the lipid‐cytokine crosstalk. However, the PGE_2_‐IFN interaction can also yield an opposite outcome in viral infection. Influenza virus infection evoked PGE_2_ production, which inhibits type 1 IFN production and suppresses apoptosis in macrophages through EP_2_ and EP_4_ receptor, leading to augmented viral infection and dissemination (Coulombe *et al*., 2014). Since macrophage apoptosis produces apoptotic vesicles containing viral antigens and type 1 IFNs can enhance antigen cross‐presentation, PGE_2_‐mediated suppression of apoptosis and inhibition of type 1 IFNs also suppressed macrophage antigen presentation and subsequent T‐cell immunity. In influenza virus infection, therefore, blocking COX‐2, mPGES1 or EP_2_/EP_4_ receptors improves survival against lethal virus infection (Coulombe *et al*., 2014).

Peters‐Golden and collaborators examined signalling mechanisms downstream of cAMP in PGE_2_‐mediated inhibition of alveolar macrophages. They found that, in alveolar macrophages, the inhibition of the generation of pro‐inflammatory mediators (such as TNF‐α, MIP‐1α and LTB_4_) and increase in IL‐10 and IL‐6 production are mediated by PKA, and suppressing FcR‐mediated phagocytosis is mediated by Epac, while inhibition of bacterial killing by ROS is mediated by both PKA and Epac (Aronoff *et al*., 2005). They then showed that PGE_2_‐EP_2_‐PKA/Epac signalling interferes with the translocation of p47phox to phagosomal membrane and thus limits bacterial killing by reducing the generation of ROS (Serezani *et al*., 2007). Wall *et al*. further found that differential effects of cAMP on the production of pro‐ and anti‐inflammatory cytokines were mediated by different classes of A kinase‐anchoring proteins (AKAPs) that complex with PKA and determine the cellular localization of PKA specific to each action. Specifically, they showed that cAMP‐dependent suppression of LPS‐induced expression of TNF‐α is carried out by the AKAP95‐PKA complex that targets PKA to NF‐κB p105 (Wall *et al*., 2009). The formation of various targeting multimer complexes may help us to understand the context‐ and cell‐dependent pro‐ and anti‐inflammatory actions of PGE_2_‐EP_2_/EP_4_ receptor signalling. Peter‐Golden's group also showed that PGE_2_ inhibits macrophage maturation. Macrophages deficient in EP_2_ receptors exhibited enhanced *in vitro* maturation and EP_2_^−/−^ mice had a higher percentage of F4/80^high^/CD11b^high^ cells and greater expression of macrophage colony‐stimulating factor receptor. This inhibitory effect was also mediated through the EP_2_ receptor ‐PKA signalling pathway (Zaslona *et al*., 2012).

### PG‐cytokine crosstalk in neutrophils

Neutrophils have been traditionally regarded as innate immune cells that combat invaders in acute infection but with restricted pro‐inflammatory functions. Recent studies, however, indicate that neutrophils are capable of a vast array of pro‐inflammatory functions and also contribute to chronic inflammation (Kolaczkowska and Kubes, 2013). Indeed, in IBDs, neutrophils extensively infiltrate the mucosa and crypts of the intestine, which correlates with mucosal injury and patient symptoms (Brazil *et al*., 2013). Recent studies have shown that PGs are involved in various pro‐inflammatory functions of neutrophils and modulate inflammation. Ma *et al*. found extensive neutrophil infiltration in association with tumour development in the colon in the AOM/DSS‐induced colitis‐associated colorectal cancer model and reported that PGE_2_ was involved in this sustained neutrophil accumulation (Ma *et al*., 2015). Infiltrating neutrophils highly express EP_2_ receptors and an EP_2_ receptors agonist and TNF‐α synergistically induced the expression of various inflammation‐related genes *in vitro* in primary cultured neutrophils, which included COX‐2, IL‐6 and CXCL1, through NF‐κB. A deficiency in EP_2_ receptors or EP_2_ antagonism abolished neutrophil infiltration, suppressed the expression of inflammatory genes including CXCL1 and decreased the number of colon tumours (Ma *et al*., 2015). These findings are consistent with the preceding study by Katoh *et al*. who used the same model and found that inflammatory cells accumulate in the colon in a manner dependent on CXCR2, a receptor for CXCL1, although they suggested that these cells are neutrophilic myeloid‐derived suppressor cells (Katoh *et al*., 2013). As neutrophils produce PGE_2_ *via* COX‐2, these results together indicate that neutrophils self‐amplify their recruitment through the TNF‐α‐primed PGE_2_‐EP_2_‐NF‐κB‐CXCL1 pathway and recruited neutrophils amplify inflammation for tumour development (Figure 4C). These results have thus provided new mechanistic interpretation for epidemiological findings showing that NSAIDs prevented colorectal cancer development and progression (Thun *et al*., 1991; Rothwell *et al*., 2010) and experimental work showing that genetic deletion of COX‐2, mPGES1 or EP_2_ receptors reduced the number and size of adenomas in AOM/DSS‐ or APC^Min/+^‐induced CRC models (Oshima *et al*., 1996; Sonoshita *et al*., 2001; Nakanishi *et al*., 2008). The involvement of PGs in the above scenario may not be limited to PGE_2_. Wallace *et al*. found that PGF_2α_‐FP receptor signalling is elevated in endometrial adenocarcinoma cells and up‐regulates tumorigenic and angiogenic genes including COX‐2, FGF2 and VEGF (Wallace *et al*., 2009). They further showed that CXCL1 and CXCR2 expression was elevated in the cancer tissue and that PGF_2α_‐FP receptors signalling promotes CXCL1 expression on endometrial adenocarcinoma cells and attracts CXCR2‐expressing neutrophils (Wallace *et al*., 2009). Neutrophil‐produced TXA_2_ has recently been reported to modulate neutrophil‐dependent control of lymphocyte egress after adjuvant administration during immunization and the neutrophil spread outside the draining lymph nodes (Yang and Unanue, 2013).

The pro‐inflammatory actions of PGE_2_‐EP_2_ receptor signalling on neutrophils described above appear to be contradictory to the classic findings that PGE_2_ inhibits human neutrophil function and migration through EP_2_ receptors (Wheeldon and Vardey, 1993; Armstrong, 1995) and the recent findings that PGE_2_‐EP_4_ receptor signalling inhibits neutrophil migration *in vivo* (Mizuno *et al*., 2014). There is also a study reporting that during acute mucosal infection with *Toxoplasma gondii*, intestinal monocyte‐produced PGE_2_ controls neutrophil activation to commensal bacteria, limiting mucosal damage during acute intestinal infection (Grainger *et al*., 2013). This apparent contradiction suggests that whether PGE_2_ exerts a pro‐ or anti‐inflammatory action depends on the context of each pathology and experimental setting such as whether PGE_2_ acts alone or with cytokines and acts in which cellular background. In contrast to PGE_2_ and PGF_2α_, PGD_2_‐DP_2_ receptor signalling is implicated as another PG signalling oppositely controlling neutrophil recruitment. DP_2_ receptor ‐deficient mice were more resistant to caecal ligation and, in a puncture‐induced sepsis model, the CXCR2‐positive neutrophil accumulation was enhanced in the infections focus, the pro‐inflammatory cytokine production reduced and bacterial clearance improved (Ishii *et al*., 2012). While this finding suggests that PGD_2_‐DP_2_ receptor signalling negatively regulates neutrophil recruitment, its mechanism remains unknown.

In inflammatory conditions, neutrophils release intracellular structures composed of chromatin DNA, histones and granular proteins, which make a net‐like structure. This structure, that is, neutrophil extracellular trap (NET), functions to trap and clear bacteria. NET formation also occurs during various chronic inflammatory diseases (Kolaczkowska and Kubes, 2013). PGE_2_ inhibited NET formation in isolated neutrophils induced by PMA or rapamycin through EP_2_/EP_4_‐cAMP receptor signalling (Domingo‐Gonzalez *et al*., 2016; Shishikura *et al*., 2016), although the extent to which this mechanism operates *in vivo* remains unknown.

### PG‐cytokine crosstalk in inflammatory stroma

The stroma of chronic inflammation is not only the site where active inflammation, tissue degradation and remodelling occur but also contributes itself to inflammation, as its cell components actively evoke and perpetuate inflammatory responses, as exemplified by fibroblast‐like synoviocytes (FLSs) in RA (Bartok and Firestein, 2010) and tumour‐associated fibroblasts (TAFs) in many cancers (Kalluri, 2016). The involvement of PGs in this active process of stroma has also been reported. For example, Honda *et al*. used collagen‐induced arthritis, a model of RA, and found that deficiency in IP receptors or EP_2_/EP_4_ receptors significantly reduced the severity of arthritis, as assessed by synovial cell proliferation, inflammatory cell infiltration and joint destruction, which was accompanied by a significant reduction in the content of IL‐6 in arthritic paws (Honda *et al*., 2006). They further used cultured synovial fibroblasts and found PGI_2_ synergized with IL‐1β to amplify IL‐6 production, which was reduced by a COX inhibitor, indomethacin. Microarray analysis revealed that, in addition to IL‐6, PGI_2_‐IP receptor signalling amplified the expression of genes related to inflammation (e.g. IL‐11 and CXCL7), cell proliferation (e.g. FGF and vascular and endothelial growth factor), tissue remodelling (e.g. RANKL and ADAM8) as well as IL‐1 receptor itself. Given that PGI_2_ alone did not induce the expression of these genes, these findings indicate that PGI_2_‐IP receptor signalling functions as an amplifier of IL‐1β signalling by expression of its receptor in synovial fibroblasts to significantly augment inflammation (Honda *et al*., 2006). Consistently, Kunisch *et al*. found, by using FLSs from RA patients, that TNF‐α stimulation drove COX‐2 expression and the production of PGE_2_, which acts back on FLSs to induce IL‐6 production *via* EP_2_ receptors (Kunisch *et al*., 2009). Paulissen *et al*. found that co‐culture of primary human Th17 cells with FLSs from RA patients led to more IL‐17A production, and this autocrine IL‐17 production as well as the induction of IL‐6, IL‐8 and MMP‐1 and MMP‐3 was effectively prevented by co‐blockade of COX‐2 and TNF‐α (Paulissen *et al*., 2013).

Similar to FLSs in RA, TAFs exert pleiotropic functions in the tumour micro‐environment for progression of cancer (Kalluri, 2016). In an aforementioned work on colitis‐associated cancer, Ma *et al*. used bone marrow chimera and found that not only EP_2_ receptor‐expressing neutrophils but also EP_2_ receptor‐expressing TAFs made a significant contribution to tumour progression. They found that the positive feedback loop of PGE_2_‐EP_2_‐NF‐κB‐COX‐2 signalling also functioned in TAFs and in synergy with TNF‐α to amplify the expression of pro‐inflammatory genes such as IL‐6, tumour promoting genes such as various WNT molecules and genes for tissue remodelling such as BDNF, MMP12 and osteopontin (Ma *et al*., 2015). These studies suggest that fibroblasts play an active role in chronic inflammation and that PG signalling contributes to their function by a synergistic interaction with cytokines (Figure 4D).

Fibrosis and angiogenesis are ultimate hallmarks of chronic inflammation. PGs also play critical roles in these processes. In the bleomycin‐induced pulmonary fibrosis model, Oga *et al*. found that deficiency in FP receptors attenuated the fibrosis (i.e. reduced collagen synthesis) in this model, which was independent of TGF‐β and without influence on inflammatory responses (Oga *et al*., 2009). Consistently, PGF_2α_ enhanced collagen synthesis in lung fibroblasts *in vitro* in an additive way to TGF‐β, indicating that PGF_2α_‐FP receptor signalling exerts a pro‐fibrotic action on its own in fibrosis (Oga *et al*., 2009). In contrast, other PGs were reported to be anti‐fibrotic. For example, mice without IP signalling had augmented bleomycin‐induced pulmonary fibrosis (Lovgren *et al*., 2006). IP KO mice developed cardiac fibrosis, which was suppressed completely by coincidental deletion of TP receptors (Francois *et al*., 2005), suggesting that IP and TP receptor signalling pathways inhibit cardiac fibrosis. PGE_2_‐EP_4_ receptor signalling has been reported to prevent tubulointerstitial fibrosis in the kidney of mice subjected to unilateral ureteral obstruction (Nakagawa *et al*., 2012). These studies indicate critical roles of PGs in the activation and function of fibroblasts, but whether these effects can be modulated by the immune system and cytokines remains for further studies. Given that both type 2 cytokines (e.g. IL‐4/IL‐5/IL‐13)‐producing Th2/ILC2 cells and IL‐17/IL‐22‐expressing Th17/ILC3 cells have been demonstrated to regulate fibrosis (Barron and Wynn, 2011; Hams *et al*., 2015) and that PGs play essential roles in the regulation of immune responses as described above, it will be interesting to examine whether PGs control fibrosis through the immune system. Likewise, PG signalling functions in angiogenesis. Amano *et al*. implanted a Matrigel sponge or tumour cells in mice and found PGE_2_‐EP_3_ receptor signalling facilitates the angiogenesis associated with chronic inflammation in the sponge and tumours (Amano *et al*., 2003). A bone marrow transfer experiment indicates that EP_3_‐bearing bone marrow‐derived cells mediate VEGF expression in the stroma around the implants and the recruitment of VEGFR‐1^+^/VEGFR‐2^+^ cells to the site of angiogenesis (Ogawa *et al*., 2009).

## Conclusion

As reviewed here, substantial evidence derived from *in vitro* and *in vivo* animal model experiments now suggest that PGs are significantly involved in chronic inflammation by regulating both innate and adaptive immune cells, and gene signature analysis of clinical samples as well as GWAS of patients appears to support these experimental findings. In the processes of chronic inflammation, PGs crosstalk intimately with cytokines in various ways. One is to induce or enhance the expression of receptor(s) for the cytokines involved, which is seen in PGE_2_‐EP_2_/EP_4_ receptor signalling‐mediated induction of IL‐12β2 and IFNγ1 receptors in Th1 cells, IL‐23 receptors in Th17 cells, PGD_2_‐DP_2_ signalling‐mediated induction of ST2 and IL‐17B receptors in ILC2 cells, and PGI_2_‐IP receptor signalling‐mediated IL‐1 receptor 1 induction in synoviocytes. Another is to collaborate with cytokines, particularly, TNF‐α, to enhance NF‐κB activation and the induction of COX‐2 to self‐amplify this signalling, which is seen in inflammation driven by macrophages such as in IA and neutrophil‐driven inflammation in colitis‐associated cancer. It may not be overstated that this COX‐2‐mediated amplification mechanism operates in various chronic inflammatory diseases that exhibit a high expression of COX‐2 together with activated NF‐κB. It is clearly noted, however, that the actions of PGs and their signalling in chronic inflammation are strictly dependent on the context, for example, the types of inflammation, cytokines, cells and so on, the timing of the disease process and the sites of inflammation, which cannot easily be generalized as seen in acute inflammation. For example, while PGE_2_‐EP_2_/EP_4_ receptor signalling facilitates Th1/Th17‐mediated immune inflammation and sustains macrophage‐ or neutrophil‐driven inflammation, the same signalling pathway dampens Th2‐mediated allergic inflammation. The same is true for PGI_2_‐IP receptor signalling. Although PGD_2_‐DP_2_ receptor signalling recruits Th2 cells, eosinophils and ILC2 cells, this signalling appears to function differently during neutrophil recruitment in the caecal ligation punture model. It should be mentioned, therefore, that although we believe in great potential of PG receptor type‐selective agonists and antagonists, the application of these drugs should be carefully thought out by analysing the clinical and pathological context of each disease in the background of experimental studies. One successful example among this line may be the application of DP_2_ antagonists to asthmatic patients (Erpenbeck *et al*., 2016; Kuna *et al*., 2016). As seen there, experimental studies to date have now accumulated enough amount of information as the base and framework for identifying a clinical indication. Given the adverse effects of traditional NSAIDs, the time is right to examine the potential of such receptor‐selective molecules to manipulate chronic inflammatory diseases. In this respect, one aspect of PG studies we cannot cover in this review due to the space limit is the action of PGs in immune evasion in cancer, which is currently an active and expanding area of research. Interested readers are recommended to read a recent review (Wang and DuBois, 2018).

### Nomenclature of targets and ligands

Key protein targets and ligands in this article are hyperlinked to corresponding entries in http://www.guidetopharmacology.org, the common portal for data from the IUPHAR/BPS Guide to PHARMACOLOGY (Harding *et al*., 2018), and are permanently archived in the Concise Guide to PHARMACOLOGY 2017/18 (Alexander *et al*., 2017a, 2017b, 2017c).

## Conflict of interest

The authors declare no conflicts of interest.
